# Whole genome sequence of *Bifidobacterium longum* subsp. *infantis* JNU311 isolated from infant feces

**DOI:** 10.1128/mra.00322-25

**Published:** 2025-08-11

**Authors:** Kiyeop Kim, SeungJi Kang, Sejong Oh

**Affiliations:** 1 Division of Animal Science, Chonnam National University 34931, Gwangju, Republic of Korea; 2 Department of Infectious Diseases, Chonnam National University Hwasun Hospital 65722, Hwasun-gun, Republic of Korea; 3 Department of Infectious Diseases, Chonnam National University Medical School 65417, Gwangju, Republic of Korea; University of Maryland School of Medicine, Baltimore, Maryland, USA

**Keywords:** *Bifidobacterium longum *subsp. *infantis *JNU311, whole genome sequence, probiotic, infant feces

## Abstract

The genome of *Bifidobacterium longum* subsp. *infantis* JNU311, isolated from the feces of a breastfed infant, was sequenced. The circular genome comprises 2,610,619 bp with 59.76% GC content. Genome annotation identified 2,306 coding sequences, suggesting potential probiotic functions and industrial applicability.

## ANNOUNCEMENT

**Fig 1:**
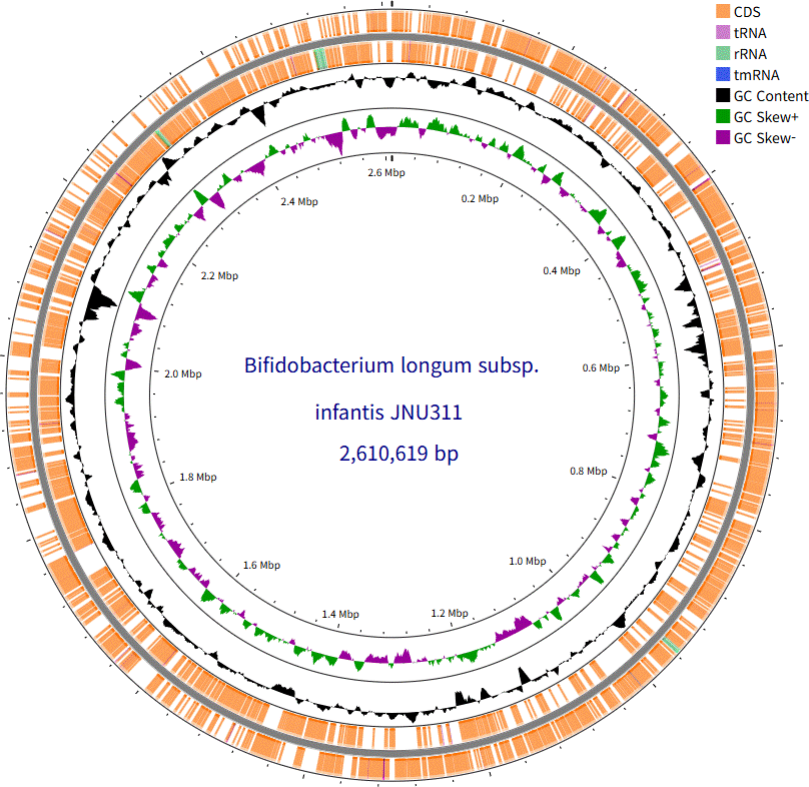
Circular chromosomal map of *B. longum* subsp. *infantis* JNU311. Marked features are shown from the periphery to the center: protein-coding sequences on the forward strand, protein-coding sequences on the reverse strand, transfer RNA (tRNA) genes, ribosomal RNA (rRNA) genes, GC content ratio, and GC skew. bp, base pairs; G, guanine; C, cytosine; CDS, coding sequence; tmRNA, transfer-messenger RNA

*Bifidobacterium longum* subsp. *infantis* dominates the gut microbiota of breastfed infants, promotes immune maturation, regulates immune balance to suppress inflammation, enhances intestinal barrier function, and increases acetate production, thereby providing various health benefits to the host (1). In this study, genome sequencing of *B. longum* subsp. *infantis* JNU311 (JNU311) was performed to explore its physiological properties and potential industrial applications. The strain was isolated from the feces of a 1-month-old breastfed infant in Gwangju, South Korea, with approval from the Institutional Review Board of Chonnam National University Hwasun Hospital (CNUHH-2025-117). Taxonomic identification was performed by amplifying the 16S rRNA gene using primers 27F and 1492R. PCR products were Sanger sequenced by Macrogen (Seoul, South Korea), and species identity was confirmed via BLASTn analysis against the NCBI 16S rRNA database. The 16S rRNA gene sequence was deposited in GenBank under accession number PV849150. The strain was initially isolated using blood-glucose-liver (BL)–neomycin-paromomycin-nalidixic acid-lithium chloride medium under anaerobic conditions at 37°C for 48 h (2). For cultivation prior to DNA extraction, the strain was grown anaerobically in BL medium (without selective agents) at 37°C for 48 h. Genomic DNA was extracted using the HiGene™ Genomic DNA Prep Kit (BIOFACT, Daejeon, South Korea). Two separate genomic DNA libraries were prepared according to the requirements of the Illumina (San Diego, CA, USA) and Oxford Nanopore Technologies (ONT) (Oxford, UK) systems. A combination of the long-read Nanopore GridION platform and short-read Illumina NextSeq2000 platform was used to generate the whole genome sequence. Illumina sequencing generated 2,730,905 paired-end reads, and Nanopore sequencing produced 103,589 long reads (397.2 Mb). After quality filtering, 2,340,533 Illumina reads (85.71%) and 75,278 Nanopore reads (381.1 Mb) were retained. The GC contents of the Illumina and Nanopore reads were 59.63% and 59.00%, respectively. The N50 of the filtered Nanopore reads was 7,471 bp. For Nanopore sequencing, a MinION sequencing library was prepared using the Nanopore Ligation Sequencing Kit (SQK-LSK114; Oxford Nanopore, UK). Genomic DNA was not intentionally sheared or size-selected prior to ONT library preparation. Low-quality bases and adapter sequences were removed from Illumina reads using Trimmomatic v0.39 (LEADING:10, TRAILING:10, SLIDINGWINDOW:4:20, and MINLEN:200) (3). Subsequently, PhiX control sequences were removed by aligning the reads against the PhiX genome using Bowtie2 v2.3.5.1 (default options) and filtering with Samtools v1.9 (4, 5). Nanopore base calling was performed using Guppy basecaller v3.1.5 (default settings), and reads with an average Phred score <7 or length <1,000 bp were filtered using NanoFilt v2.8.0 (6). Default parameters were used for all software unless otherwise specified. Genome assembly was performed using Unicycler v0.4.8 (default parameters) by combining filtered NextSeq2000 and GridION data (7). The final assembled genome of strain JNU311 was 2,610,619 bp long with a GC content of 59.76% (Table 1, Fig. 1). The sequencing coverage was 517.725× for Illumina sequencing and 137.924× for Nanopore sequencing. The assembled draft genome consisted of a single contig. Genome completeness was assessed using BUSCO v5.2.2 with the actinobacteria_class_odb10 database (8). The results revealed 322 (90.4%) complete BUSCOs. Of these, 319 (89.6%) were complete and single copy, while three (0.8%) duplicated genes. Of the 356 BUSCO groups searched, four (1.1%) were fragmented and 30 (8.4%) were missing. Circularity of the genome was confirmed using Unicycler v0.4.8, which detects overlapping ends during hybrid assembly (7). The assembled genome was not rotated to a specific gene. The genome was annotated using the NCBI Prokaryotic Genome Annotation Pipeline, which identified 2,306 coding sequences, 58 tRNAs, and 12 rRNAs. These results suggest that JNU311 holds potential for probiotic applications, and further studies are needed to explore its functional roles and clinical benefits.

**TABLE 1:** Genome features of *B. longum* subsp. *infantis* JNU311^*a*^

Feature	Value
Genome length (bp)	2,610,619
GC content (%)	59.76
Illumina sequencing depth	517.725
Nanopore sequencing depth	137.924
CDS sequences	2,306
tRNA genes	58
rRNA genes	12

^*a*^ bp, base pair; G, guanine; C, cytosine; CDS, coding sequences; tRNA, transfer RNA; and rRNA, ribosomal RNA.

## Data Availability

The whole genome sequence has been deposited in NCBI GenBank under the accession number CP166499. The associated BioProject, BioSample, and 16S rRNA gene accession numbers are PRJNA1142219, SAMN42954375, and PV849150, respectively. The raw sequencing reads are available in the NCBI Sequence Read Archive (SRA) under accession numbers SRR33364941 (Illumina) and SRR33364940 (Nanopore).
